# A listener preference model for spatial sound reproduction, incorporating affective response

**DOI:** 10.1371/journal.pone.0285135

**Published:** 2023-06-14

**Authors:** George Moiragias, John Mourjopoulos

**Affiliations:** Audio and Acoustic Technology Group, Department of Electrical and Computer Engineering, University of Patras, Rio Patras, Achaia, Greece; Bialystok University of Technology, POLAND

## Abstract

This work presents results and models for listener preference to music delivered via different spatial reproduction formats, here via mono, stereo and multichannel (5.1-ch) reproduction. Although this problem has been researched in the past, the current work introduces an elaborate multistage experimental procedure which considers the contribution of listener-specific emotional responses (valence and arousal) to his/hers Overall Listening Experience (OLE). The test procedure registers the individual listener preference and familiarization with the content of each test audio sample. A spatial envelopment metric, extracted directly from each audio signal sample is utilized as attribute for the perceived differences between the 3 different systems. This attribute, along with listener content preference (for each music sample) and his/hers affective response attributes are combined into linear regression model(s) which can predict the dominant trends for rating OLE. A novel linear tree approach is also proposed which highlights additional associations between the attributes within this multidimensional space. Comparative performance analysis shows that the proposed linear tree approach also achieves improved predictions for OLE ratings.

## Introduction

Today, music can be delivered to the listeners via a wide range of formats and options. Towards a more holistic analysis of the the listener experience and preference, here the effect of different spatial audio reproduction formats, i.e monophonic (mono), stereophonic (stereo), multichannel (surround), is examined noting that such preference can be only partially correlated to established objective metrics for the system’s properties [1–3]. As with many other aspects of audio system performance, a growing body of recent research concerns methods that can also describe and predict the listener’s experiences incorporating individual preferences for the content, i.e. the music material [4–6].

Models suitable for listener individualization, adaptation to specific target groups and task-specific applications in audio engineering can be traced back to the general architecture for quality assessment introduced by Blauert and Jekosch [7] and modified later by Raake and Blauert [8]. Recently, for quality assessment of events, scenes and sounds more elaborate perceptual features and cognitive functions are incorporated in computational models of binaural hearing and perception, suitable for engineering applications [9, 10] aiming to accommodate the important stages of perceptual inference and knowledge. Complementary to such models, listener evaluations via semantic sensory descriptors [11], or explicit reference stimuli [12], also provide a framework that can register and predict the listener experience and preference. For example, the listener preference was assessed via a metric of the system’s basic audio quality (BAQ) [13], which rated both spatial and timbral system quality but excluded any listener subjective preference rating. This is also the case for the widely used consensus-based lexicon tests [14, 15], which exclude all pleasure-related (“hedonic”) descriptors. To register such factors, the established semantic sensory descriptor methods have been recently augmented via affective and pleasure-related (hedonic) descriptors based on Continuous Quality Scales (CQS) and Hedonic Scales (LHS) [16]. Furthermore, the established Quality of Experience (QoE) method [17], was redefined via the term Overall Listening Experience (OLE) [18], specifically within the context of listening to music via an audio system. Through OLE assessment, the listeners are asked to take into consideration every factor that influences their preference, as if they were in a real-life scenario.

In [18, 19] the participants were divided through a statistical analysis into “audio quality likers” and “song likers” and it was found that the OLE ratings of the first group had a stronger correlation to audio system limitations (in this case, reduced bandwidth), while the ratings of the second group showed a high correlation with the content of the music excerpt. A later experiment investigated the impact of different spatial audio reproduction systems (mono, stereo and 5-channel surround) on OLE [20]. It was found that OLE ratings were influenced both by the content of the music excerpts and to a lesser extent by the reproduction system. Some participants based their preference mostly on the content, while others on the system used for reproduction. Similar trends for OLE were also found from evaluations of 3D audio systems [4] and from comparisons of different reproduction systems [5, 6]. In a previous work of the authors [21], a simple regression model for OLE prediction of binaurally recorded music excerpts in three different formats (mono, stereo and surround) was introduced and the results indicated that it is the preference for the content of the music excerpts that dominated on OLE formation, whilst a lesser impact was registered by the perceptual factors (in this case only Envelopment was considered) introduced by the audio system.

Semi-empirical approaches for predicting OLE, from model parameters directly obtained from listening test data have also evolved, e.g. Schoeffler et al. [22] suggested a generic model for predicting OLE ratings, in which a reference and a signal under test were compared. A cumulative link model (CLM) was applied to the results of an experiment [20] and the OLE was modelled as the linear combination of two parameters: the preference of the reference stimulus (preference for the stimulus’ content) and the reproduction system. It was found that content preference was more correlated with OLE than the reproduction system, although the model’s relatively low predictive power implies that several additional mechanisms affecting OLE may be also involved.

Here, affective listener response is considered as a potential additional factor contributing towards the individualized overall preference. As is known [23–25], a significant aspect of listener preference to the music is clearly formed due to his/her affective response. Thus, towards a more holistic methodology in audio engineering, this work formalizes a listener model which can also predict preference to audio stimuli via elicitation of emotional (affective) response that is both content and listener dependent [7], but also considers the contribution of the audio system [11], in this case via its spatial reproduction properties. The proposed model evolves from an extended and novel test framework, conducted in 3 distinct stages: the first aiming to establish the listener profile with respect to content (i.e. a baseline for the individual’s cognitive state with respect to the music samples), the second eliciting listener responses with respect to the 3 different spatial reproduction arrangements (i.e. the perceptual attributes of audio reproduction) and the third stage, recording listener emotional response and OLE. Here, the extension of the OLE prediction model is initially formulated via a linear regression combining the cognitive, perceptual and emotional attributes. The test results indicated a more complex relationship between users’ responses and OLE to the one predicted by such linear regression. For this, a novel linear tree approach is also introduced, which is shown to improve the predictive accuracy of such a listener model and reveals possible additional intuitive mechanisms of OLE formation, that have not been reported before. An additional novel finding of the proposed procedure is that the perceptual and differentiating attributes between the 3 tested audio formats, could be accurately extracted from the test signals (i.e. the different music samples), thus facilitating both the test procedure and also the utilization of the resulting model.

The paper is organized as follows: Section **Analysis Method** introduces the analysis framework employed in this paper. The **Experimental Method** section describes the experimental procedure and section **Results** reports the experiment results. Section **Discussion** outlines the results and addresses the required future work. Finally, a summary of this work is given in the **Conclusion** section.

## Analysis method

### Assessment attributes and feature space of the listener model

The listening test assessed several attributes and consisted of 3 different sessions, which were conducted in periods separated by 10 days in order to reduce bias due to listener short-term memory.

- In the first session (**S1**), the subjects evaluated their preference (CP) and familiarization (F) for the content of all music samples, in this case presented via headphones in a mono format.
- In the second session (**S2**), the music samples were presented via mono, stereo and surround loudspeaker set-up. The subjects rated the perceived spatial audio quality using 2 attributes: Envelopment (Env) and Clarity (Cl).
- In the last session (**S3**), the subjects rated their Overall Listening Experience (OLE) and evaluated the evoked emotions. As with S2, the material was again presented via mono, stereo and surround loudspeaker set-up.

The total set of assessed attributes are listed in Table 1, along with the type, range of their values and the scales used for their assessment. A more detailed analysis of these attributes is presented in subsection Listening Sessions. A categorical variable was introduced, named System (*S*), describing the reproduction format and taking the value *M* if the format was mono, St if the format was stereo and Sr if the format was surround.

**Table 1:** Rated attributes of the experimental procedure and their properties. The hedonic responses, Content Preference and Overall Listening Experience, are registered using the Modified Labeled Hedonic Scale (MLHS), the perceptual attributes using the Spatial Quality Scale (SQS) and the emotions of the listeners using the Affective Slider of Arousal (ASA) and the Affective Slider of Valence (ASV).

Listening Sessions	Rated Attributes	Rating Scale	Type	Range of Values	System (S)
S1	Content Preference (CP)	MLHS	interval	[-100,100]	M
	Familiarization (F)	five-star Likert	ordinal	{1,2,3,4,5}	M
S2	Envelopment (Env)	SQS	interval	[-100,100]	M, St, Sr
	Clarity (Cl)				M, St, Sr
S3	Arousal (A)	ASA	interval	[-100,100]	M, St, Sr
	Valence (V)	ASV	interval	[-100,100]	M, St, Sr
	Overall Listening Experience (OLE)	MLHS	interval	[-100,100]	M, St, Sr

The attributes affecting OLE form a *Z*^*j*^ feature space, where *j* is the number of all possible attributes that may have an effect on OLE. An OLE predictive model can be seen as the task of combination of such attribute dimensions into a multidimensional hyperplane. As will be presented in subsection OLE Model Trees, via the linear tree approach, such high dimensionality for OLE can be considered as a structure of individual subspaces, each combining different attributes.

### Modeling emotions

Affective models have been employed mainly for assessing listener responses related to music content and to a lesser extend for assessing audio system factors [26, 27]. Such models are successfully incorporated in the majority of Music Emotion Recognition (MER) tasks [28–31]. In general, emotion rating is based on two main approaches: the categorical (discrete) emotion models [32–34] and the dimensional models of emotions [35–37], although newer music-related emotion models have also emerged [38]. The most commonly used model in MER is the circumplex model of affect (or Valence-Arousal model) proposed by Russell [36], where emotions exist on a plane along independent axes of arousal, ranging from high to low and valence (pleasure), ranging from positive to negative.

It has to be noted that there is a distinction between perceived and induced emotions. Perceived emotions are related with the perception of emotions expressed by the music content, while induced emotions are those evoked to the listener [39, 40]. Most of MER researches are either assessing the perceived emotion of subjects or do not state which one they are evaluating (see [41] for a review), providing no insight about the affective states of subjects. Only by evaluating the induced emotion, the affective state of a subject during a listening procedure can be assessed.

Vuoskoski et al. [42] investigated the applicability of discrete, continuous and music-specific models in the assessment of music-induced emotions. It was found that the continuous model [35] was the most efficient and after a principal component analysis (PCA), that its three-dimensions could be merged to two. Song et al. [43] showed that circumplex could more efficiently describe music-mediated emotions than categorical model and that induced emotional responses had a higher uncertainty level, indicating the different affective states evoked to the listeners by the same music sample. Taking into account the above analysis, the affective states of the assessors during a listening procedure are assessed by asking them to evaluate their induced emotion, using the circumplex model.

### Modeling envelopment

George et al. [44, 45] developed an unintrusive model for predicting the sensation of envelopment from multichannel sound recordings, based solely on signal related features. In their work, downmix algorithms, bandwidth limitations and low bit-rate coding were also applied to the multichannel signals and the accuracy of the model showed a high correlation (*r* = 0.9) between predicted and actual scores obtained from listening tests. This model is described by the equation:

$$\begin{array}{cc} \text{Envelopment}= & 51.75+0.0016R_{raw}+4.31ASD-27.19I_{OB60}I_{OB150}-0.23KLT_{V1}I_{OB60}\\ & +0.13KLT_{V1}CCA_{log}+\epsilon \end{array}$$ (1)

where *R*_*raw*_ is the spectral roll-off of the mono downmixed signal, *ASD* is the area of sound distribution around listener, *I*_*OB*60_ is the mean value of the average octave-band interaural cross correlation (*IACC*) at horizontal head rotation of 60° and 300°, *I*_*OB*150_ is the mean value of the average octave-band *IACC* at horizontal head rotation of 150° and 210°, *KLT*_*V*1_ is the percentile variance of first eigenchannels of the Karhunen-Loéve Transform (*KLT*) [46] of the signals, *CCA*_*log*_ is the log_*e*_ value of the centroid of the distribution of eigenchannels’ direction and *ϵ* is the error term of the model. More features were initially used (e.g. *BFR*: the ratio of average energy in rear channels to front channels), which were excluded after an iterative regression analysis. Further information about the features and the way they are computed can be found in [45].

Starting from such approach, a linear model (Envelopment Model) was implemented throughout this work, in order to predict the mean value of the Env ratings of each music sample obtained during S2. It has to be noted that in the context of the present paper the signals were presented in only three spatial formals (mono, stereo, 5-channel surround), without bandwidth limitations or low bit-rate coding, so the features coefficients will be different to those in Eq (1). Thus, here the Envelopment Model is described by the following equation:

$$\begin{array}{cc} Env= & a_{0}+a_{1}R_{raw}+a_{2}ASD+a_{3}I_{OB60}I_{OB150}+a_{4}KLT_{V1}I_{OB60}+a_{5}KLT_{V1}CCA_{log}\\ & +a_{6}BFR+\epsilon \end{array}$$ (2)

Correlation of this model to listener Env ratings are presented in subsection S2-Spatial Audio Quality.

### Modeling OLE

The Overall Listening Experience (OLE) can be modeled by the appropriate combination of the attributes that can potentially affect it e.g those in Table 1. These models can be evaluated on their ability to predict accurately the dependent variable, in this case OLE, as is registered by listening tests. Here, three metrics for the evaluation of the models were used:

Adjusted *R*^2^, a metric indicating the percentage of variance of OLE that the model explains, given by the following equation:

$$\begin{array}{c} R^{2}=1-\frac{\sum_{i=1}^{n}{\left({\text{OLE}}_{i}-\hat{{\text{OLE}}_{i}}\right)}^{2}}{\sum_{i=1}^{n}{\left({\text{OLE}}_{i}-\bar{\text{OLE}}\right)}^{2}} \end{array}$$ (3)

$$\begin{array}{c} \text{Adjusted}\;R^{2}=1-\frac{\left(1-R^{2}\right)\left(n-1\right)}{n-k-1} \end{array}$$ (4)

where $\hat{{\text{OLE}}_{i}}$ are the values of OLE predicted by the model for each music sample *i*, $\bar{\text{OLE}}$ is the mean of OLE listener ratings, *n* is the number of the samples used for fitting model and *k* is the number of the independent variables employed.

Root mean square error (*RMSE*):

$$\begin{array}{c} RMSE=\sqrt{\frac{\sum_{i=1}^{n}{\left({\text{OLE}}_{i}-\hat{{\text{OLE}}_{i}}\right)}^{2}}{n}} \end{array}$$ (5)

Mean absolute error (*MAE*):

$$\begin{array}{c} MAE=\frac{\sum_{i=1}^{n}|{\text{OLE}}_{i}-\hat{{\text{OLE}}_{i}}|}{n} \end{array}$$ (6)

#### OLE linear models

Using ordinary least square regression, a simple linear model can be implemented for such task and its goodness of fit is evaluated by reporting the adjusted *R*^2^. For example, in [4], a linear model was implemented for predicting OLE, described by Eq (7).

$$\begin{array}{c} \text{OLE}=b_{0}+b_{1}\text{CP}+b_{2}S+\epsilon , \end{array}$$ (7)

where CP is the Content Preference, *S* indicates the reproduction format of each sample, *b*_1_, *b*_2_ are the coefficients of the variables, the standardized values of whose indicate the strength of association and *ϵ* is the error term of the prediction. A variation of Eq (7), that takes under consideration perceptual attributes accounting for the Spatial Audio Quality, can be:

$$\begin{array}{c} \text{OLE}=b_{0}+b_{1}\text{CP}+b_{2}\text{Env}+b_{3}\text{Cl}+\epsilon , \end{array}$$ (8)

In this way, OLE is not associated to the reproduction system itself, but only to its dominant qualitative attributes perceived by the listener. By introducing now the emotions of assessors induced during the listening procedure, such a linear model becomes:

$$\begin{array}{c} \text{OLE}=b_{0}+b_{1}\text{CP}+b_{2}\text{Env}+b_{3}\text{Cl}+b_{4}A+b_{5}V+\epsilon \end{array}$$ (9)

where *A*,*V* are the reported Arousal and Valence ratings respectively. Thus, Eq (9) presents the proposed potential simplified relationship between OLE and listener induced emotions delivered via a different audio system spatialisation. Such a novel approach relates the subject’s Overall Listening Experience or aesthetic judgement [11, 47], via the dominant binaurally perceived attributes, i.e. the envelopment (Env), due to audio spatial presentation, along with the individual listener’s cognitive bias, i.e. the prior content preference (CP) and also the dominant emotional factors (*A*, *V*).

#### OLE model trees

Given that the limited dataset size of this type of listening test did not allow for complex machine learning methods to be used, a variation of the decision tree algorithm was introduced. Decision trees are hierarchical models composed of decision rules that are applied recursively to partition the feature space in order to predict the target variable [48]. Each tree has two types of nodes: branch nodes and leaf nodes. In the branch nodes the feature space is divided by the selected rule and in the leaf nodes the final predictions are made based on the subspace defined by the corresponding parent nodes. In the leaves of linear tress, linear regression models are implemented [49, 50]. Here, the partition of the dataset was based on [51].

In the case of OLE prediction, a split is performed for each sample of each attribute, dividing the feature space in two subspaces of $\hat{j}$ ≤ *j* dimensions and two linear models are created for each subspace. The subspaces created correspond to the left and right node of each branch node. In this recursive procedure, a weighted loss function (WLF) is computed for each child node described by the following equation:

$$\begin{array}{c} WLF_{k_{a,s}}=\frac{n_{k}}{n}\sqrt{\frac{\sum_{i=1}^{n_{k}}{\left(OLE_{{k_{i}}_{a,s}}-\hat{OLE_{{k_{i}}_{a,s}}}\right)}^{2}}{n_{k}}} \end{array}$$ (10)

where *a* is the attribute used for the split, *s* is the value of this attribute, *k* ∈ *r*, *l* is an index corresponding to either the subspace of right child node (*r*) or left child node (*l*), *n* is the number of the samples of the parent node, *n*_*k*_ is the number of samples in the corresponding child node and $\hat{{\text{OLE}}_{{k_{i}}_{a,s}}}$ are the OLE values, predicted from the corresponding linear model. A total loss function (TLF) is computed for the children nodes, given by the equation:

$$\begin{array}{c} TLF=WLF_{r_{a,s}}+WLF_{l_{a,s}} \end{array}$$ (11)

TLF is computed for each possible split of a branch node and the split with the lowest TLF is selected. If the selected TLF is lower than the root mean squared error (RMSE) of the parent node’s model, the split is performed, otherwise the tree stops to grow and no children nodes are created.

**Fig 1:**
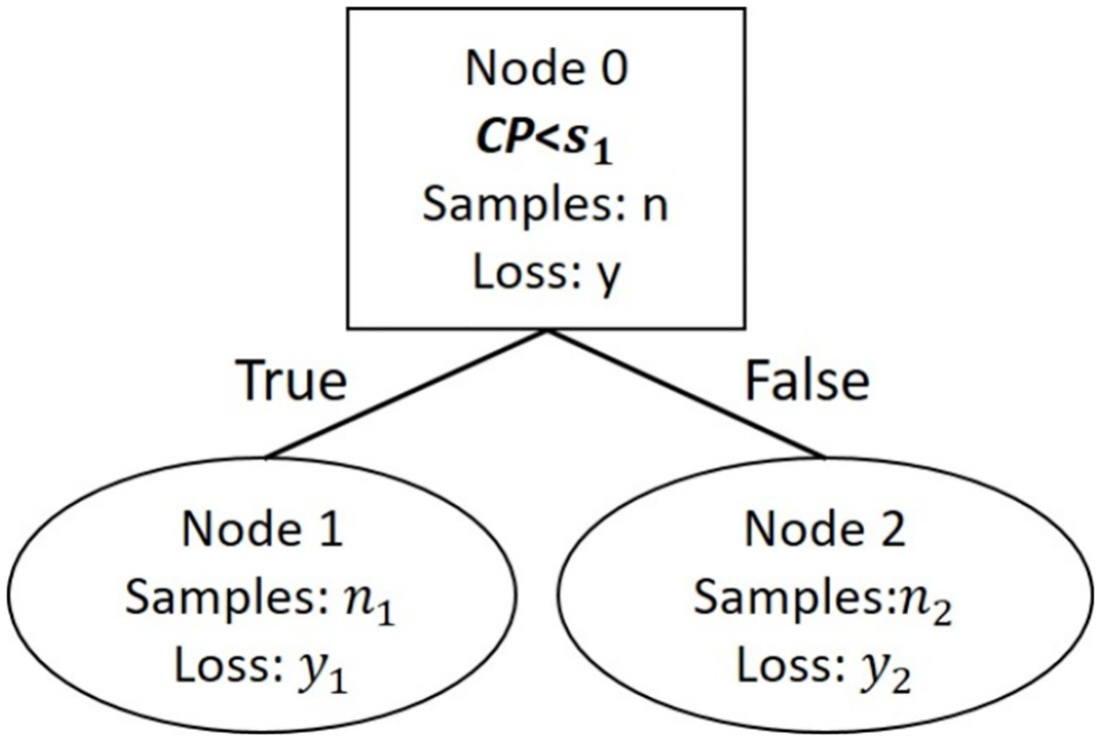
Example of linear tree architecture. Linear Tree with one branch node and two generated leaf nodes using Content Preference (CP) ratings to split the dataset. Node 1 contains the assessments with CP values less than *s*1; Node 2 has CP values greater or equal to *s*1.

A simple example for a one level OLE prediction linear tree, incorporating 3 attributes (CP, Env, *A*) and using CP to split the dataset, is shown in Fig 1, along with Eq (12) which describes each leaf node and the corresponding condition (Eq (13)).

$$\begin{array}{c} \hat{\text{OLE}}=\{\begin{array}{cc} b_{00}+b_{01}\text{CP}+b_{02}\text{Env}+b_{03}A, & \text{CP}<s_{1}\\ b_{10}+b_{11}\text{CP}+b_{12}\text{Env}+b_{13}A, & \text{CP}\geq s_{1} \end{array} \end{array}$$ (12)

$$\begin{array}{c} \frac{n_{1}}{n}y_{1}+\frac{n_{2}}{n}y_{2}<y \end{array}$$ (13)

*n*_1_ and *n*_2_ are the number of samples in Node 1 and Node 2 respectively, *y*_1_ and *y*_2_ are the root mean squared errors for Node 1 and 2 respectively and *y* is the root mean squared error of the parent node (Node 0). In the above case, the subspaces of each leaf node have the same dimensions as the parent node (*Z*^3^), meaning that all three attributes have an effect on OLE prediction, although the effect of these attributes may differentiate within each subspace e.g. *b*_01_ ≠ *b*_11_. However, if the coefficient of an attribute in a leaf node is not statistically significant and/or has a value near zero, then it will be excluded from the corresponding node. For example in Eq (12), if *b*_02_ is not included in the model, then the feature space of Node 1 is *Z*^2^, indicating that OLE is affected only by CP and *A* when CP has a value lower than *s*1. This data-oriented approach may verify intuitive aspects or even provide counter-intuitive mechanisms for OLE formulation.

## Experimental method

The Research Ethics Comittee of the University of Patras waived the need for approval for the current work (protocol number: 12204), as all data were analyzed anonymously and the assessors did not provide personal information. In addition, all the assessors provided verbal consent for registering their responses, prior to the experimental procedure, witnessed by the authors. The study did not include minors.

### Participants

In total, 25 participants, mostly students and researchers at the University of Patras completed all three sessions of the experiment. The average age of the participants was 27.48 years with a standard deviation of 9.5 and seven participants were female. Twelve participants had never participated before in a listening test, 9 had participated 1–5 times, 1 had participated 6–10 times and 3 had participated in more than 10 listening tests.

### Stimuli

Fifteen song excerpts with a duration of almost 20 seconds were selected for the experiment (see S1 File) originally from high resolution digital audio recordings mixed both in multichannel (5.1 or 5.0) and stereo format (2.0). For the mono version of each sample, the corresponding stereo file was down mixed by adding the two channels and all audio excerpts were loudness–normalized to -22 LUFS according to [52]. In addition, an informal loudness normalization task was performed by the experimenters, so as to ensure that all music samples presented in any spatial format were of similar loudness. For samples whose surround format contained a discreet LFE channel, this was added to the respective stereo and mono audio files after the loudness-normalization. In total, 45 samples (15 songs*3 systems) were created.

### Materials and apparatus

All the listening tests were conducted in a listening room which conforms to ITU-R BS.1116 [53], containing a 5.1 installation, based on non-specialist 2-way speakers (KRK Rokit 5) and a Crystal Audio (THX-10SUBT) subwoofer, arranged according to [54], which were used for playback of the samples for sessions S2 and S3 (see below). The system reproduction level was calibrated with a sound meter to 85 dBA SPL for full scale pink noise adjusting each speaker separately so that the measured SPL at listeners’ head position was identical. The LFE channel level was almost 10 dBA higher than the rest loudspeakers. As described below, the session S1, was reproduced via Ultrasone S-Logic Pro 650 headphones and in all experiments, the assessors registered their responses via the webMUSHRA graphical interface [55]. The 3 different listening sessions described below, were separated by a break of approximately 10 days so that any familiarity with content was not retained.

### Listening sessions

**Fig 2:**
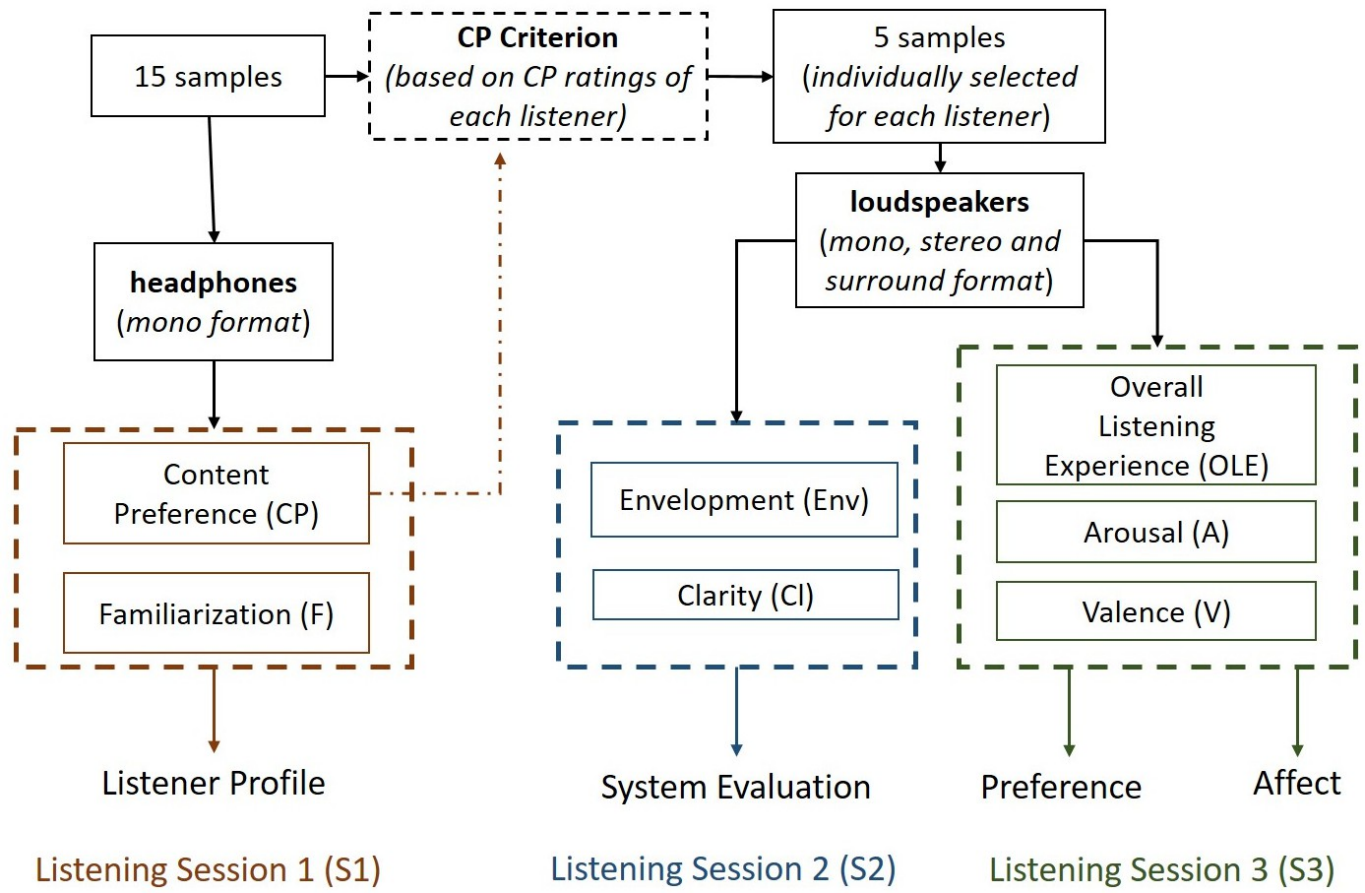
Overview of the experimental procedure.

An overview of the experimental procedure is shown in Fig 2. This procedure was identical for each participant and consists of three different listening sessions. Initially, all 15 music samples were reproduced via headphones in a mono format and the listeners were asked to register their preference (CP) and familiarization (F) solely for the music content of the samples (Listening Session 1). Based on the individual CP ratings (CP Criterion, see subsection CP Criterion) 5 samples were selected for each participant for the two remaining listening sessions. The individually selected music samples were presented via loudspeakers in three different conditions (mono, stereo and surround) for Listening Session 2 and 3. During Listening Session 2, listeners evaluated system-related perceived differences via assessing the Envelopment (Env) and Clarity (Cl) of the spatially reproduced music samples. During Listening Session 3 they assessed Overall Listening Experience (OLE), induced Arousal (*A*) and induced Valence (*V*). It has to be noted that in each session (described in detail in the following sections) the assessment order of each attribute was randomized to avoid order effects.

#### S1-Listener profile

At the beginning of this session, an instruction page was presented to the participants explaining the purpose of the test and they were informed to rate their preference for each music sample taking into consideration everything that is important to them, as if they were in a private listening condition, by answering the question “How much do you like each music item?” as in [20]. These ratings thus are called Content Preference (CP) ratings for the remainder of the paper.

**Fig 3:**
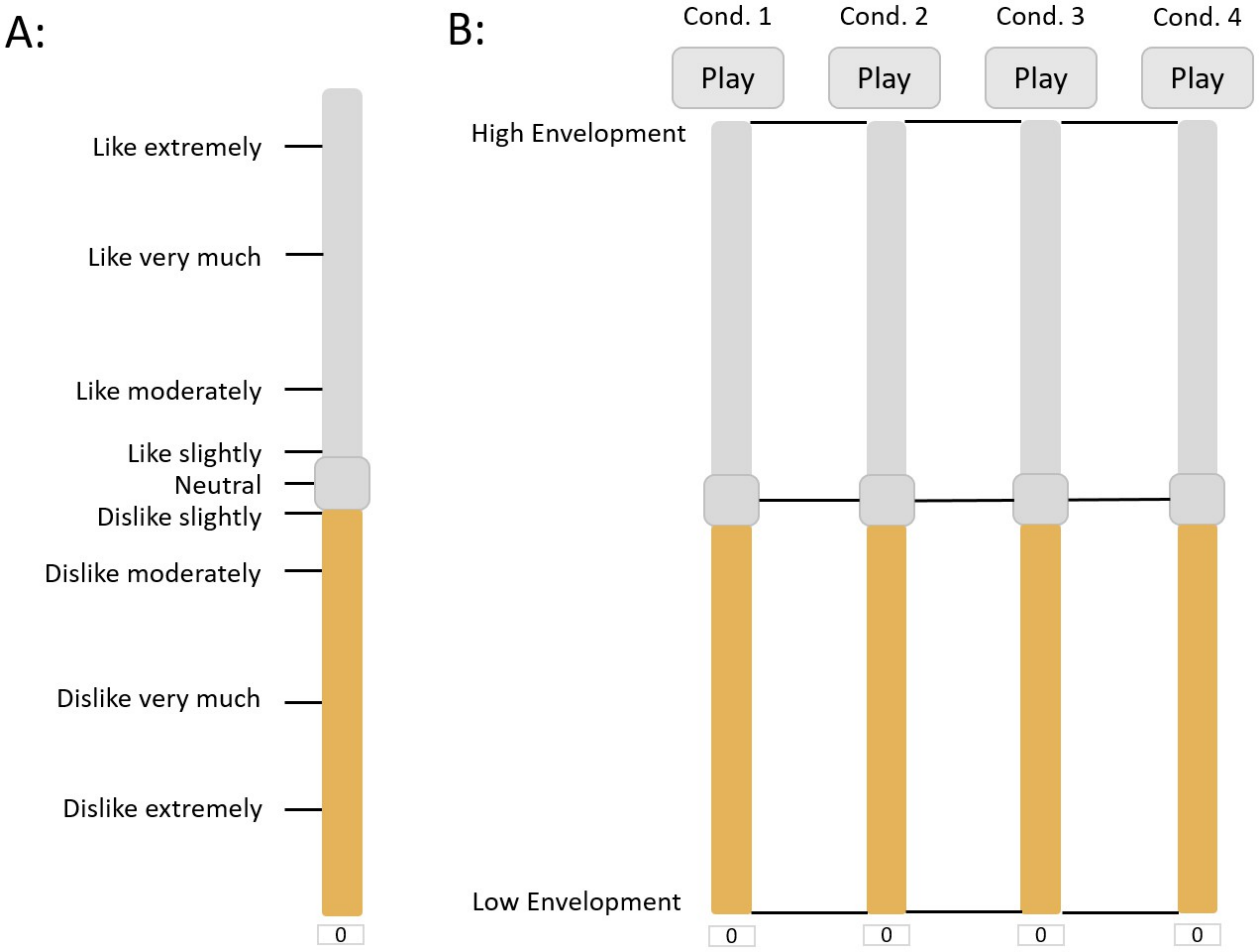
Assessment scales for listening Sessions S1 and S2. A: Modified Labeled Hedonic Scale (MLHS) for the assessment of preference. B: Session S2 user interface for the assessment of Envelopment for four conditions of one music sample, using the Spatial Quality Scale (SQS).

For the content preference ratings, a modified Labelled Hedonic Scale (MLHS) [56] was used (see Fig 3A), which is continuous with 9 verbal labels slightly asymmetrically distributed around Neutral (zero). The semantic labels of MLHS are not evenly spaced accros the scale, as the hedonic intervals between adjacent categories are unequal. This is is a variation of the Labelled Hedonic Scale [16] with the two most extreme labels removed, in order to avoid the so-called compression effect. Neutral corresponds to value 0, the upper limit of MLHS to 100 and the lower limit to -100. Before the main content preference rating session, the participants were asked to rate their preference for 4 music samples in order to become familiarized with the GUI and the procedure. These 4 ratings were not taken under consideration in the subsequence analysis.

Additionally, the listeners were informed that they would be asked to evaluate their familiarization with each sample and fill a questionnaire. For the evaluation of familiarization a five-star Likert scale was used with the stars labeled as “Not at all”, “Not a lot”, “A little”, “Much”, “Very Much”. Note that all questions and instructions were translated to Greek since all the assessors were native Greek speakers.

#### CP Criterion

5 music samples were selected for each participant to assess for Listening Session 2 and 3 (see Fig 2) to avoid listener fatigue and loss of concentration during the assessment procedure, as it would require 45 assessments per attribute (15 music samples * 3 different formats), if all listeners assessed all music samples. The procedure for the selection of 5 samples for each listener was based on the methodology introduced in [20], in which music samples were individually selected depending on listener’s CP during S1, in order to assure that the complete range of the individual listener content preferences was represented by the test samples. This selection criterion is thereafter named CP Criterion.

Five rating groups were generated from the preference ratings of each assessor, as shown in Table 2. One music sample from each of the five groups was selected as stimulus for the next sessions, thus each assessor had to evaluate the remaining attributes of 5 different samples. If for a participant, one group contained no music sample, then a music sample with the closest to this group rating was selected.

**Table 2:** Content Preference (CP) rating groups based on Modified Labeled Hedonic Scale (MLHS) employed for the the individualized selection of samples for listening Session 2 and 3.

Ratings Group	MLHS Labels
1	Lower limit to “Dislike very much”
2	“Dislike slightly” to “Dislike very much”
3	“Dislike slightly” to “Like slightly”
4	“Like slightly” to “Like very much”
5	“Like very much” to Upper limit

#### S2-System evaluation

Before the assessment of Env and Cl, a training page was generated which contained the definition and an example of the corresponding attribute, so that the assessors could become well familiar with them. The definition of Env was the following: “Envelopment defines the extent to which sound encloses (‘wraps’) and engages the listener”. The definition of Cl was: “Clarity defines how clearly the listener can discern the individual audio sources.” A continuous scale was used for the evaluation of each attribute, ranging from -100 to 100 (Spatial Quality Scale). High values of the scale indicate high values of the corresponding attribute and vice versa. In each page, only one music sample was presented and one quality attribute was evaluated by the participants. For each music sample, one randomly selected spatial format (mono, stereo, surround) was presented twice without the assessors being aware of it, hence four conditions of the music excerpt had to be evaluated (see Fig 3B). This allowed a post-screening of the assessors via the measurement of the reliability of their ratings (see S2 File).

#### S3-OLE and affect

The assessment of Overall Listening Experience was performed using the MLHS scale (Fig 3A). Each page presented only one musical sample in three different spatial formats and again listeners were guided to take into consideration everything that is important to them. These ratings reflect the Overall Listening Experience preference for the music samples, taking also into account the specific spatial rendering and format.

**Fig 4:**
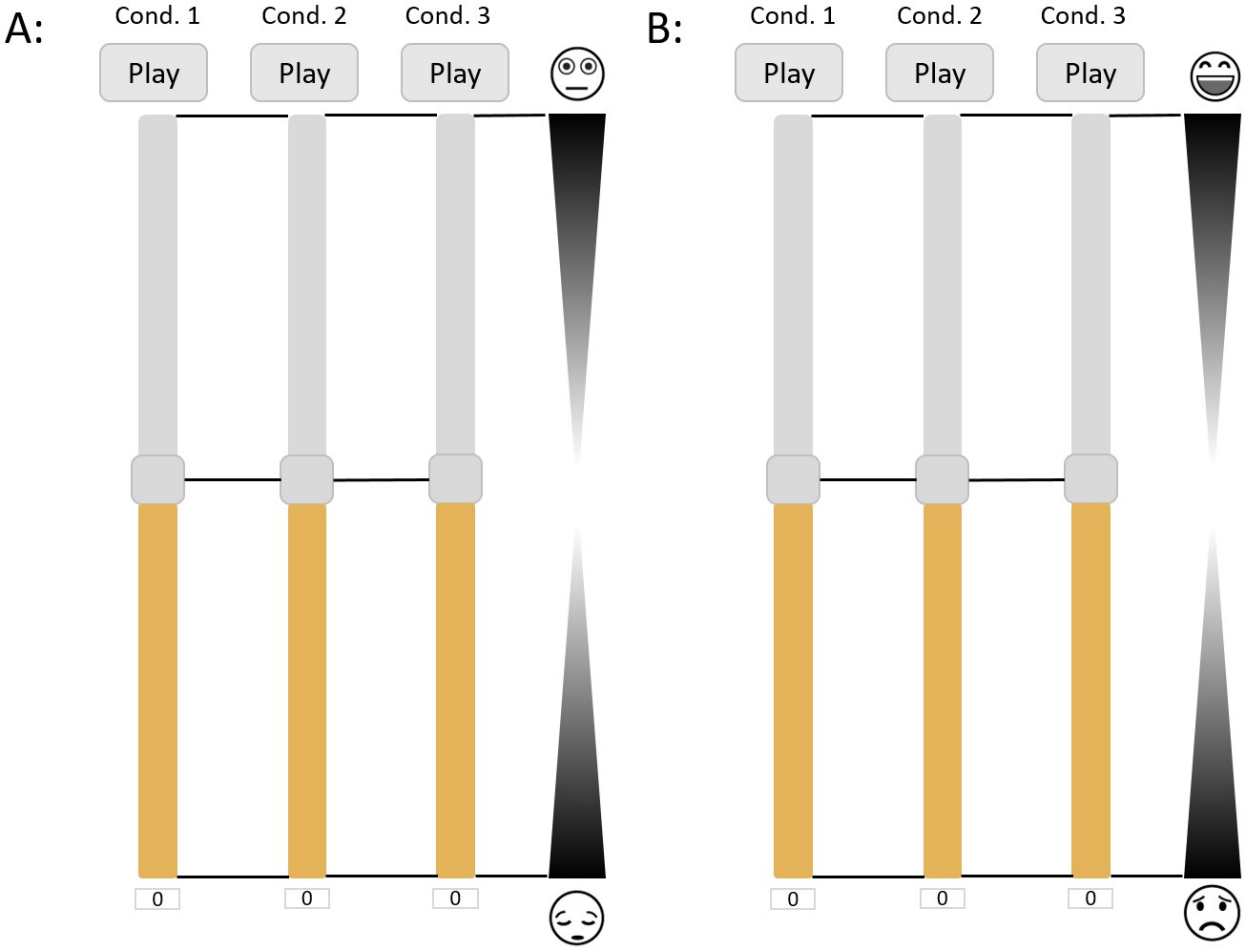
Session S3 user interface for A: Arousal and B: Valence assessment using ASA and ASV respectively.

In the second part of S3 the participants had to annotate the emotions that each spatially reproduced music sample evoked in them. The assessment of participants’ induced emotion was performed via a variation of a non-verbal continuous scale named Affective Slider (AS) [57], one for Arousal (ASA) and one for Valence (ASV), as is shown in Fig 4. AS was found to be highly correlated with Self Assessment Mannequins (SAM) [58], which are mainly utilized in emotions assessment, indicating that AS can provide valid emotion ratings based on the circumplex model of affect (see subsection Modeling Emotions). Before the actual rating, the participants could freely ask for further clarifications on the use of the above-mentioned scales.

## Results

A description of the statistical analysis implemented in the following subsections is presented in S3 File.

### S1-Listener profile

As explained before, the session S1 assessed the preference for the music content (CP) and the listeners’ familiarization (F) with it. To examine if the familiarization of a music sample has an effect on preference, Kendall’s *τ* coefficient between these two variables is reported, due to the categorical nature of familiarization ratings.

**Fig 5:**
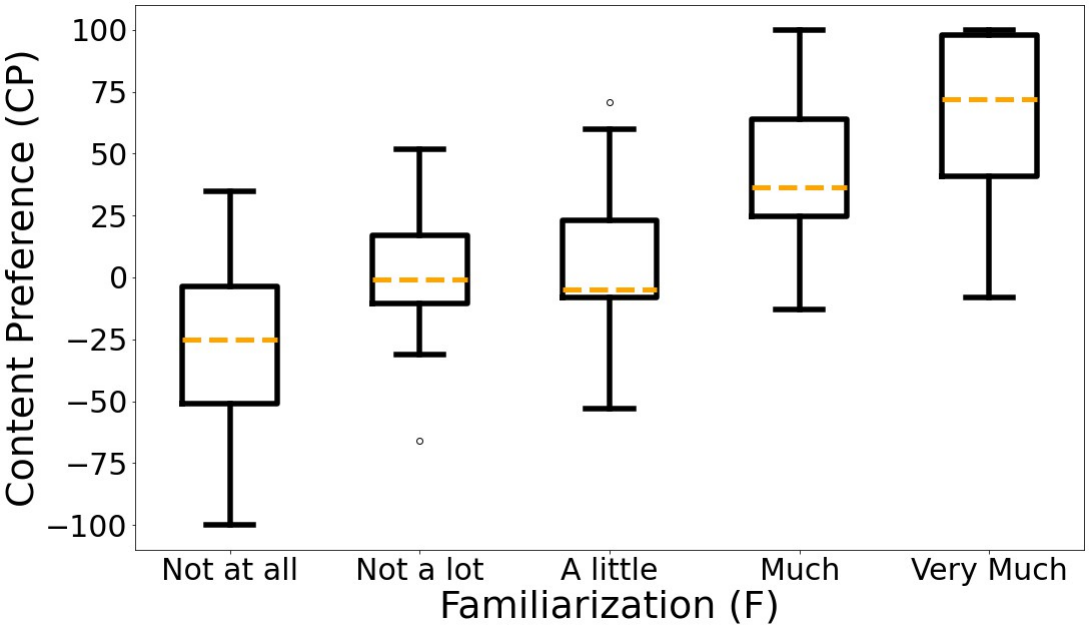
Session S1 CP ratings boxplot distributions for each familiarization category. The more familiar a music sample is, the more probable is its CP rating to be higher.

The boxplot distributions of CP ratings for each F value are shown in Fig 5. The correlation between F and CP was *τ* = 0.580 (*p* <.001). Hence, for the tested music samples familiarization was related to content preference, as it was expected, and is not further considered to the rest of the analysis.

### S2-Spatial audio quality

**Fig 6:**
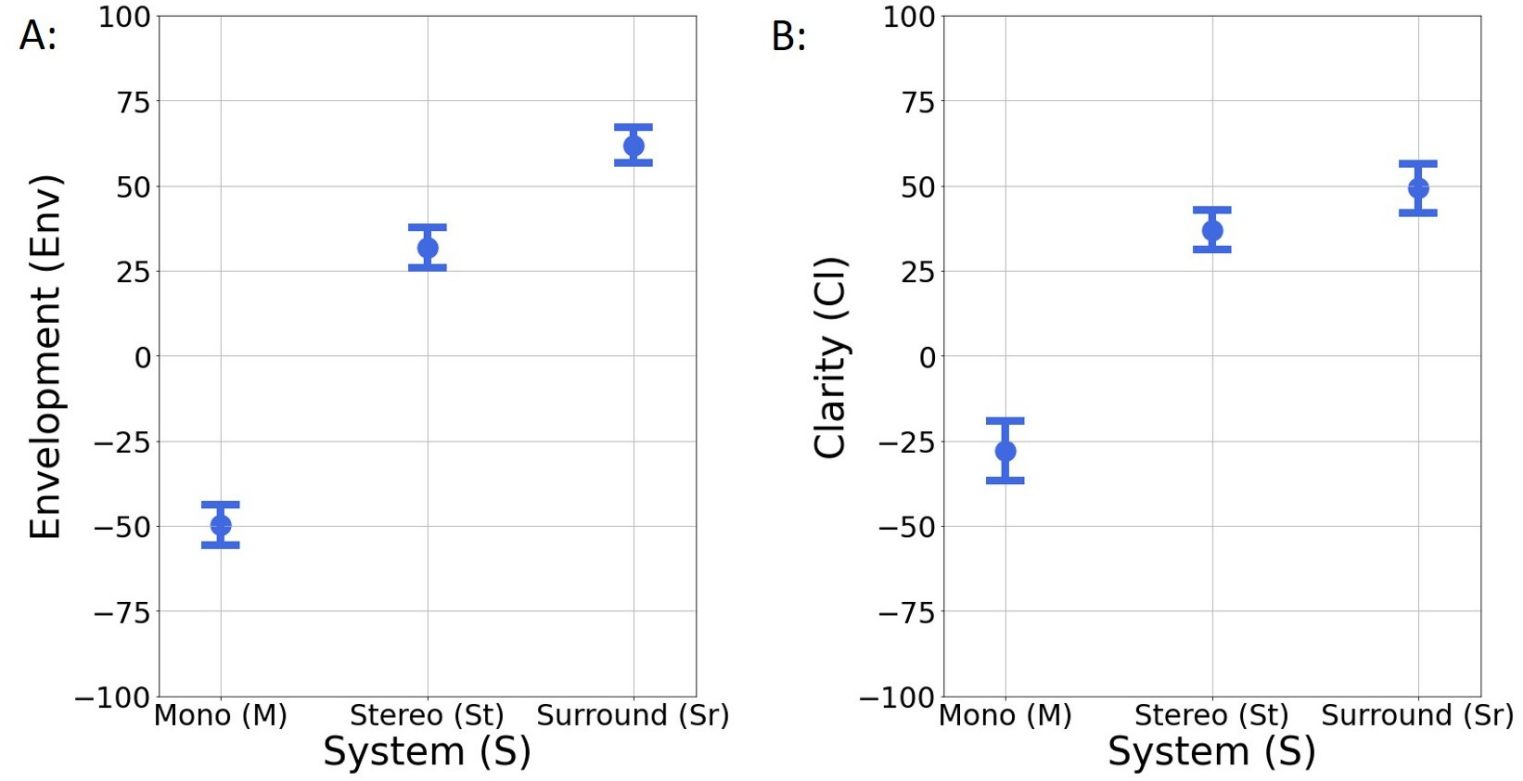
Mean values and 95% confidence intervals for Session S2 ratings. A: Envelopment and B: Clarity ratings for each reproduction system: Mono (*M*), Stereo (St) and Surround (Sr).

Two attributes were evaluated by the assessors during S2: Envelopment (Env) and Clarity (Cl) and the results are shown in Fig 6. To determine which attribute is more suitable to describe the different audio reproduction systems, one-way ANOVA was performed for each of the above attributes to determine if there were statistically significant differences between the ratings for each set-up (*M*, St and Sr).

For Env, the differences between *M*, St and Sr are significant (*F*(2, 312) = 392.911, *p* <.001) with a strong effect size *η*^2^ = 0.716. For Cl, due to violation of homogeneity of variance, a Welch ANOVA analysis was performed and the differences between *M*, St and Sr were found to be significant (*F*(2, 202.06) = 100.345, *p* <.001) with effect size *η*^2^ = 0.443. To examine the means differences between each pair of the groups (St-*M*, Sr-*M* and Sr-St), post hoc tests were performed. The Tukey test was used for the ANOVA analyses and the Games-Howell test for the Welch ANOVA analysis. The results are given in Table 3 along with the corresponding values of Cohen’s *d*.

**Table 3:** Pairwise comparisons between each reproduction system for Envelopment (Env) and Clarity (Cl) ratings based on Cohen’s d.

Attributes	Pairs of Set-ups		
	St-*M*	Sr-*M*	Sr-St
Env	d = 2.6 (p<.001)	d = 3.8 (p<.001)	d = 1 (p<.001)
Cl	d = 1.7 (p<.001)	d = 1.8 (p<.001)	d = 0.36 (p = .026)

The large effect sizes of the ANOVA analyses for Env and Cl, indicate that these attributes are strongly associated with the reproduction format and can be used as its descriptors, as was also purposed by previous researchers [14, 15]. The large *d* reported for every pair of loudspeakers set-up for Env indicates that this attribute differentiates strongly between *M*, St and Sr format, while the attribute of Cl does not differentiate a lot between St and Sr format (due to small effect size).

Considering the above and to simplify the analysis, only the attribute of Env will be subsequently used to describe the spatial quality for the rest of the paper, which is in accordance with a previous research by Francombe et al. [59], in which the increased listener envelopment (immersion) of the reproduced sound field had been established as the spatial attribute with stronger impact on listeners’ preference.

Utilizing Eq 2, the Envelopment values predicted by the proposed Envelopment Model ($\hat{\text{Env}}$) are given by Eq 14, while its standardized version is described by Eq 15.

$$\begin{array}{c} \hat{Env}=26.109+1.509KLT_{V1}CCA_{log}-0.1988KLT_{V1}I_{OB60}-19.293BFR \end{array}$$ (14)

$$\begin{array}{c} \hat{Env}=0.413KLT_{V1}CCA_{log}-0.977KLT_{V1}I_{OB60}-0.266BFR \end{array}$$ (15)

The adjusted *R*^2^ of Envelopment Model is 0.843. The absolute values of the coefficients in Eq 15 capture the significance of each feature on predicting Envelopment. The product of *KLT*_*V*1_ and *I*_*OB*60_ had the largest impact on predicting Envelopment, whilst *BFR* had the smallest. Due to the high variance explained by the Envelopment Model, subjects’ ratings of Env in S2 can be substituted by objective signal-derived features, thus enabling an engineering approach that can describe directly the Spatial Audio Quality and hence to some extent, also OLE.

### S3-OLE and affect assessment

#### OLE assessment

**Fig 7:**
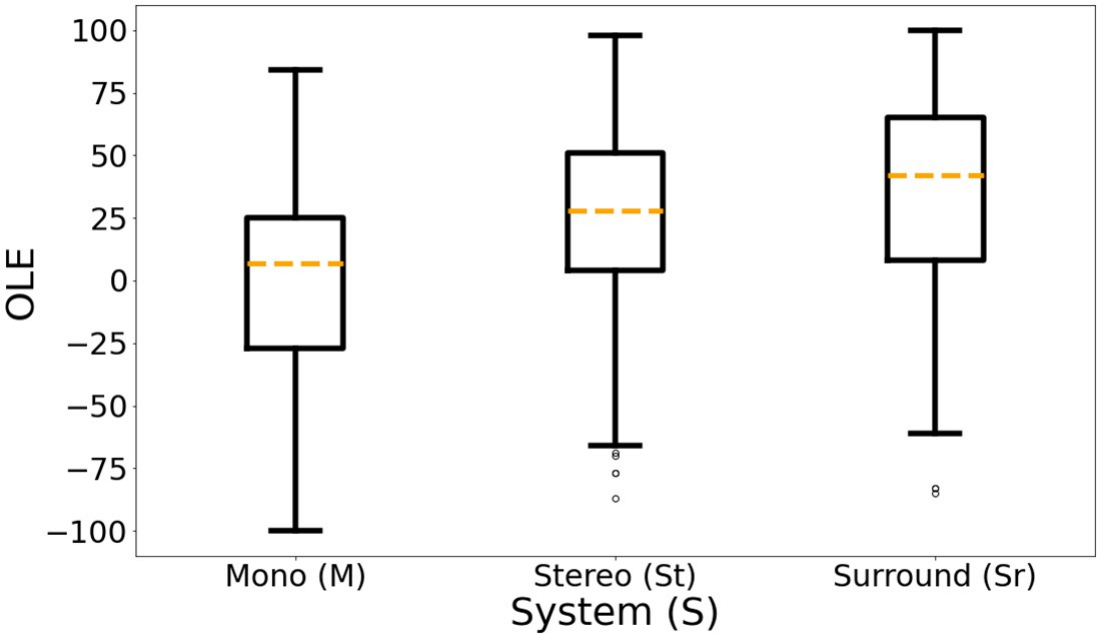
OLE ratings boxplot distributions for each reproduction system: Mono (M), Stereo (St) and Surround (Sr).

The boxplot distributions of OLE ratings for each system obtained during S3 are shown in Fig 7. To investigate the impact of the system on OLE, a one-way ANOVA was calculated, followed by a post hoc Tukey test. A significant effect of system was found (*F*(2, 312) = 18.358, *p* <.001) with effect size *η*^2^ = .105, followed by a Tukey test and the report of Cohen’s d (see Table 4).

**Table 4:** Pairwise comparisons between each reproduction system for Overall Listening Experience (OLE) ratings based on Cohen’s d.

Attributes	Pairs of Set-ups		
	St-*M*	Sr-*M*	Sr-St
OLE	d = 0.54 (p<.001)	d = 0.82 (p<.001)	d = 0.3 (p = .075)

Although OLE ratings for both St and Sr are statistically significant different of those for *M* (evoking higher OLE ratings), the difference between St and Sr is not significant, indicating that potentially the Sr system does not further increase OLE ratings. This saturation effect of OLE ratings, for increasing number of reproduction channels, has been also reported in [4, 59].

#### Valence assessment

**Fig 8:**
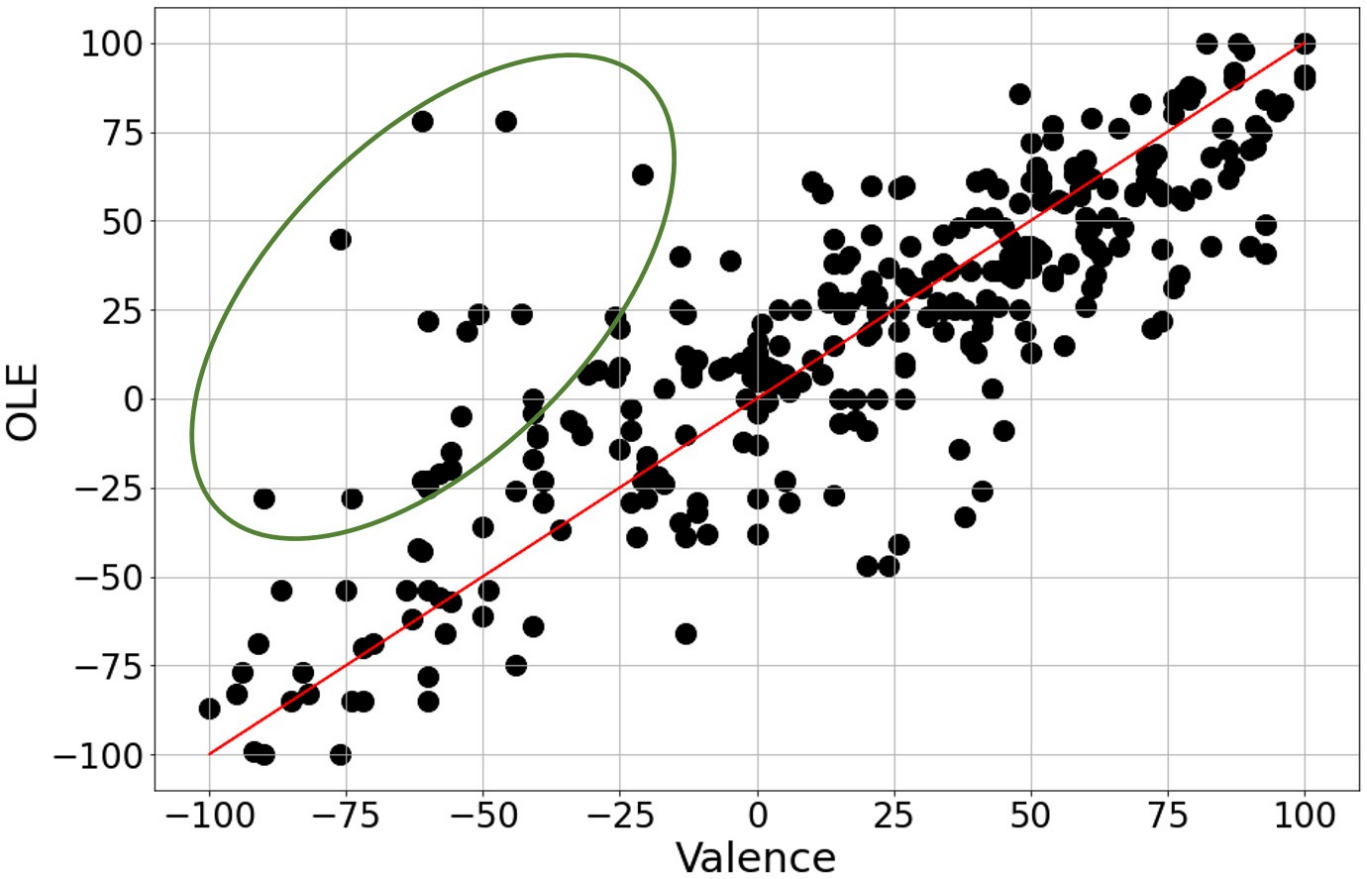
Session S3 ratings results for OLE versus valence. Inside the green ellipse are the samples with high OLE, but low Valence ratings. The red line represents the ideal perfect correlation between OLE and Valence.

Induced Valence and preference (in this case defined by the term OLE) have been found to be highly correlated [60, 61], as it is likely to describe the same attribute i.e. Valence stands for the evoking pleasure to a assessor listening to a music sample via a spatial reproduction format, whilst OLE stands for the overall listening experience assigned by the listener to the same stimulus. Fig 8 shows the registered induced valence and OLE ratings and it becomes apparent that these two attributes were interpreted in the same manner by the assessors, due to their high correlation. This was also verified by verbal feedback of the assessors after the end of the session S3. Pearsons’ r between V and OLE was equal to 0.829 (*p* <:001). It should be noted, that there is a small amount of large discrepancies between Valence and OLE ratings, indicated by the green ellipse, which may be due to the incorrect registration of the perceived rather than the induced emotions. In these cases, music samples with high OLE ratings were assigned negative V values due to the sad emotion perceived by the assessors (e.g. paradox of “enjoyable sadness” [62]). Therefore, the attribute of induced Valence is not further considered in the following analysis.

#### Arousal assessment

**Fig 9:**
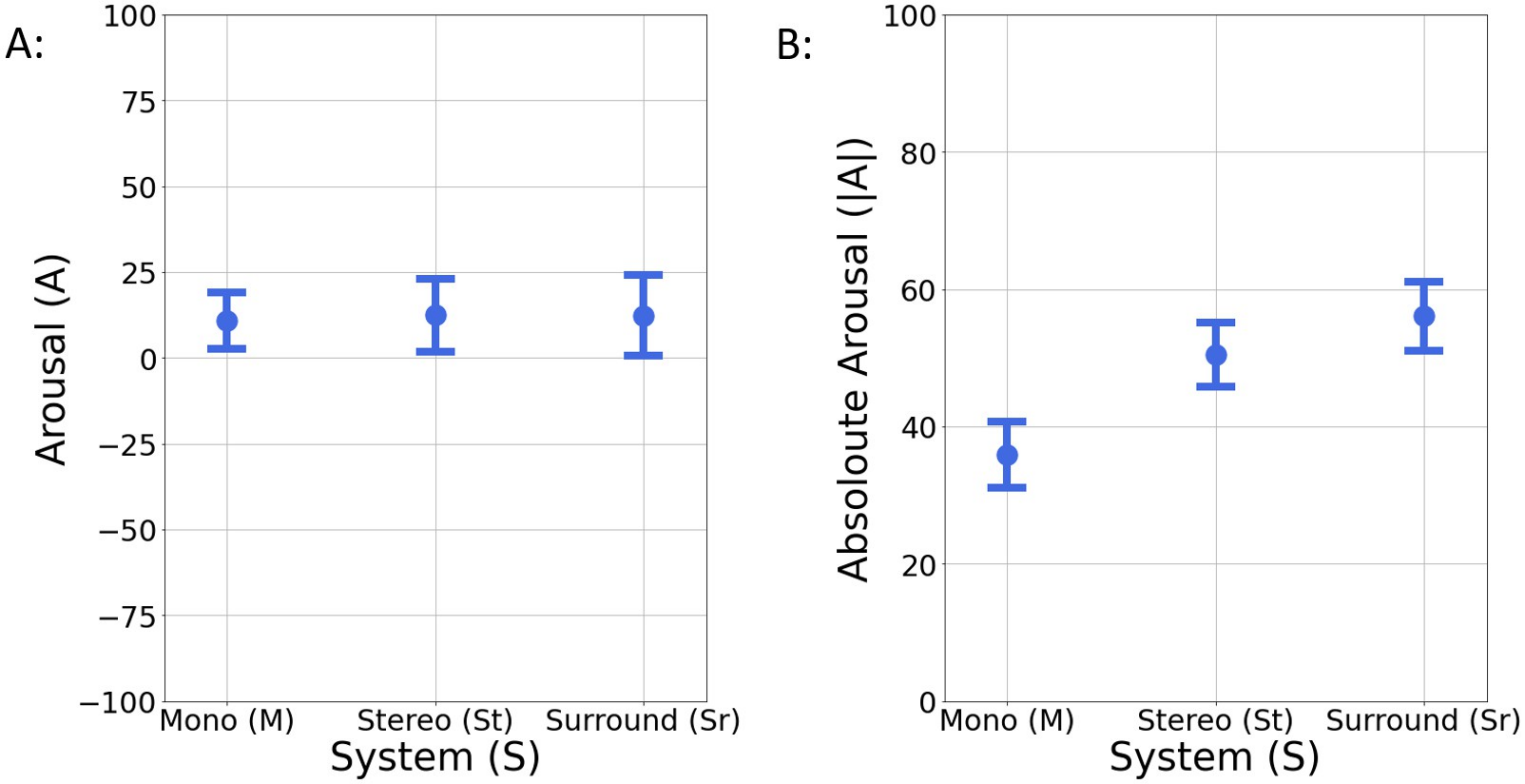
Mean values and 95% confidence intervals for Arousal ratings. A: Arousal and B: absolute Arousal ratings for each reproduction system: Mono (*M*), Stereo (St) and Surround (Sr).

The effect of reproduction system and stimulus on *A* ratings was analyzed via two-way ANOVA and a significant effect of stimulus was found (*F*(14, 270) = 15.683, *p* < 0.001) with effect size $\eta_{G}^{2}=.448$, while the effect of system was not significant (*F*(2, 270) = .032, *p* = .969) with effect size $\eta_{G}^{2}=.000$, as the interaction of system and stimuli (*F*(28, 270) = .491, *p* = .987) with $\eta_{G}^{2}=.048$. This indicates that the emotional response related to *A* depends primarily on the stimulus and not on the different reproduction format. The independence between *A* and system is illustrated in Fig 9A.

**Fig 10:**
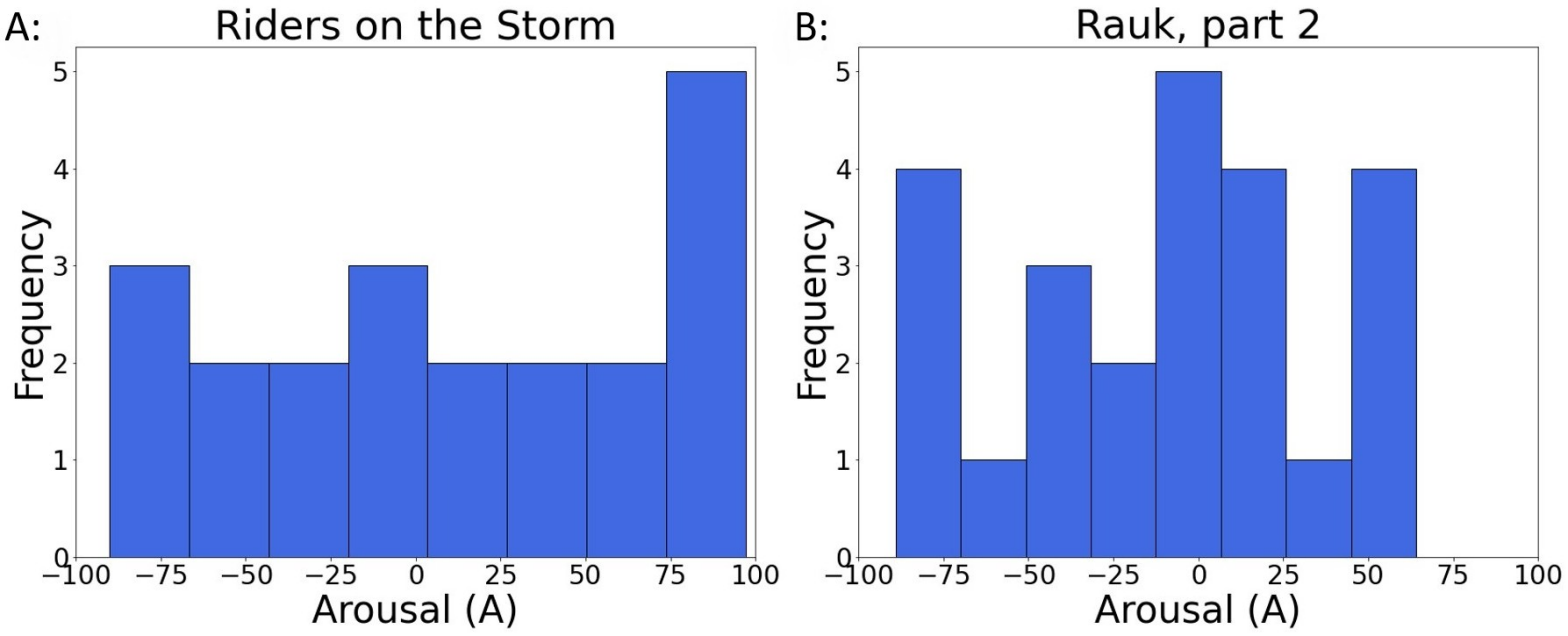
Arousal ratings distributions for two samples. A: “Riders on the Storm” and B: “Rauk, part 2”. The high variances of these distributions with ratings across all the range of *ASA* scale indicate that the induced affective states of the listener are highly individual.

However, it was also noted that some music samples had uniform like distributions, indicating that Arousal-related emotions induced through a listening procedure were highly dependent on the individual subject. For example, as it is illustrated in Fig 10, depending on the subject, the sample “Riders on the Storm” had both high and low *A* ratings and a similar trend was observed for the sample “Rauk, part 2”.

Taking this into account, a one-way ANOVA was run on the absolute values of *A* ratings (see Fig 9B), in order to examine if system has an effect on inducing more intense emotional states (meaning the extremes of *ASA* scale). From this perspective, the results showed that there is a significant effect on system for |*A*| (*F*(2, 312) = 18.264, *p* <.001) with effect size *η*^2^ = .105. To examine the means differences between each pair of the groups, a Tukey post hoc test was performed and the results are depicted in Table 5.

**Table 5:** Pairwise comparisons between each reproduction system for absolute Arousal (|A|) ratings based on Cohen’s d.

Attributes	Pairs of Set-ups		
	St-*M*	Sr-*M*	Sr-St
|*A*|	d = 0.59 (p<.001)	d = 0.80 (p<.001)	d = 0.23 (p = .235)

The values of effect sizes reveal that there is a medium to large effect of system on |*A*|, for St-*M* and Sr-*M* comparisons, but no statistical significant differences were found when St format was compared to Sr. Thus, both St and Sr reproduction formats lead to more intense Arousal-related emotions compared to mono (*M*).

### OLE model prediction results

#### Linear models

The results obtained by the implemented linear models for predicting OLE (see subsection OLE Linear Models) are now presented. These models should predict OLE listener ratings from the rated values of the respective attributes. For each model the standardized values of the attributes employed were considered to illustrate their corresponding effect, along with adjusted *R*^2^, utilizing only the statistically significant coefficients. The first model (Model 1) using Eq (7), was the following:

$$\begin{array}{c} \hat{OLE}=0.730\text{CP}+0.370\text{Sr}+0.235\text{St} \end{array}$$ (16)

where the variables Sr and St get the value 1, if the corresponding stimulus was reproduced in surround or stereo format respectively, otherwise they are assigned to 0 and $\hat{\text{OLE}}$ are the predicted OLE values of the model. The adjusted *R*^2^ of Model 1 was 0.635.

Taking into account the results of subsection S2-Spatial Audio Quality and using Eq 8, Model 2 was implemented, substituting variables Sr and St with signal-derived $\hat{\text{Env}}$:

$$\begin{array}{c} \hat{OLE}=0.737\text{CP}+0.297\hat{Env} \end{array}$$ (17)

The adjusted *R*^2^ of Model 2 was 0.619.

Finally, based on the affect analysis of subsection Arousal Assessment and using Eq (9), Model 3 was fitted, which also incorporates the absolute values of Arousal (|*A*|). The adjusted *R*^2^ of Model 3 was 0.626 and is described by Eq (18).

$$\begin{array}{c} \hat{OLE}=0.734\text{CP}+0.271\hat{Env}+0.094|A| \end{array}$$ (18)

In all the above models, CP has the highest standardized value, indicating that the preference of the content has the greatest impact on OLE. System related attributes (*Sr*,*St* and $\hat{\text{Env}}$) also affect OLE but to a lesser degree. Finally, the standardized coefficient of |*A*| was very low, thus it does not have a crucial impact on OLE, compared to CP and $\hat{\text{Env}}$.

#### OLE model trees

**Fig 11:**
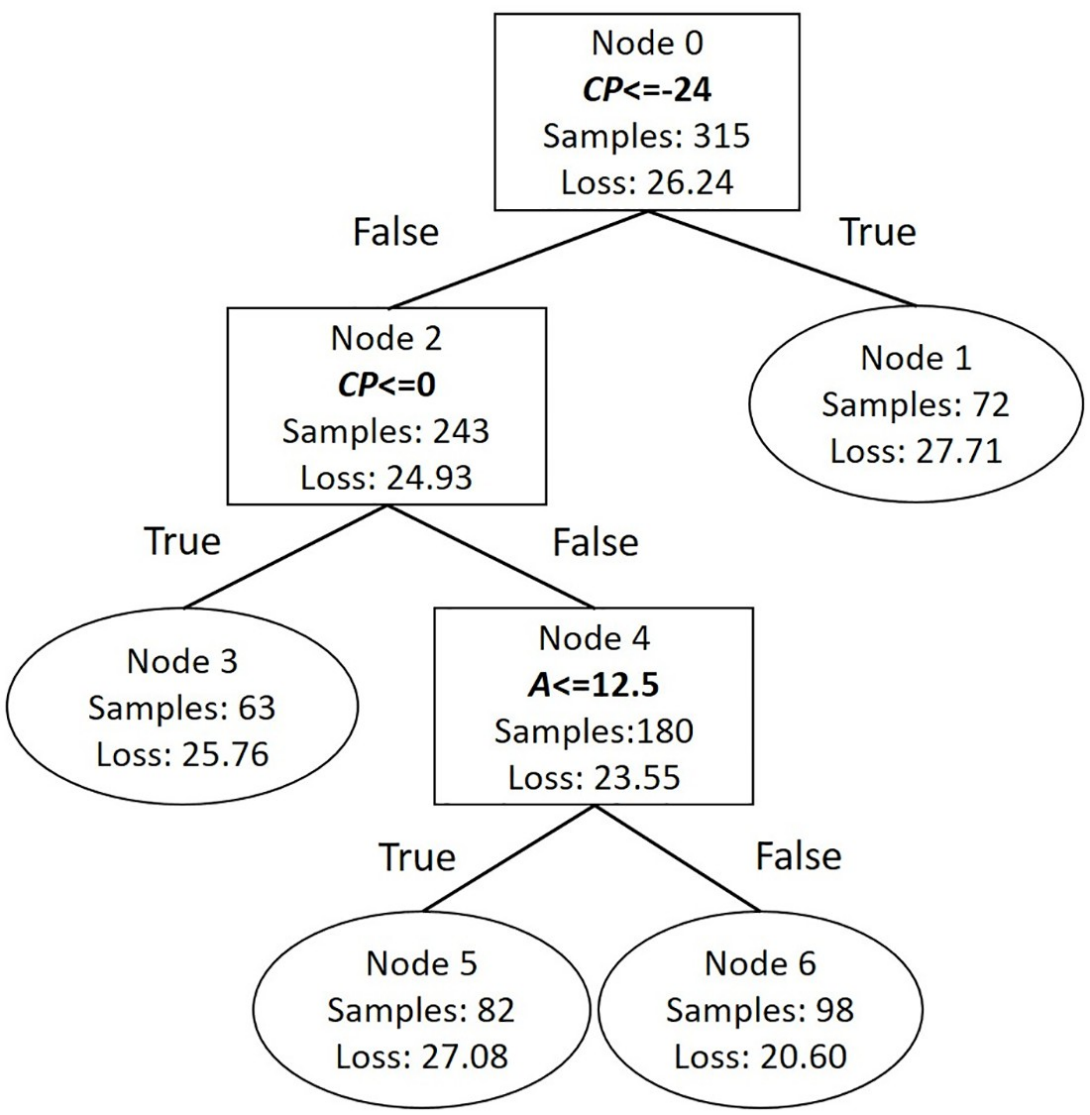
Linear Tree Model architecture resulted from the proposed linear tree algorithm. Nodes 0,2 and 4 are branch nodes, in which the feature space is divided according to the corresponding attribute e.g. in Node 4 the feature space is divided based on the Arousal (*A*) ratings. Nodes 1,3,5 and 6 are the leaf nodes, in which linear regression models were implemented.

For simplicity, a three-level linear tree was fitted to the obtained data, using only three attributes that were found to be related with OLE: CP, $\hat{\text{Env}}$ and *A* (see Linear Models). The architecture of the resulting model (Linear Tree Model) is shown in Fig 11.

In each leaf node (Node 1, 3, 5 and 6) a linear model was fitted to the corresponding samples. The equations describing each model are the following:

$$\begin{array}{c} Node1:\hat{OLE}=0.689\text{CP}+0.245\hat{Env} \end{array}$$ (19)

In this case, Eq 19 predicts OLE for samples that their content was greatly disliked (CP ≤ − 24). The resulted OLE mainly depends on CP and to a lesser extent on $\hat{\text{Env}}$.

$$\begin{array}{c} Node3:\hat{OLE}=0.430\text{CP}+0.459\hat{Env}+0.231A \end{array}$$ (20)

Eq 20 predicts OLE for samples that their content was disliked (−24< CP ≤0). CP and $\hat{\text{Env}}$ had almost the same impact on OLE, while *A* affected OLE to a lesser extent.

$$\begin{array}{c} Node5:\hat{OLE}=0.345\text{CP}+0.313\hat{Env}-0.197A \end{array}$$ (21)

Eq 21 predicts OLE of samples whose content was liked (CP >0) and had low *A* ratings. In this case, OLE was positively related to CP, $\hat{\text{Env}}$ and negatively to *A*.

$$\begin{array}{c} Node6:\hat{OLE}=0.576\text{CP}+0.351\hat{Env}+0.177A \end{array}$$ (22)

Eq 22 predicts OLE of samples whose content was liked (CP >0) and had high *A* ratings (*A* > 12.5). In this case, OLE was positively related to CP, $\hat{\text{Env}}$ and *A*. It has to be noted that the models’ coefficients presented above were standardized and only the statistically significant (*p* <.05) coefficients were incorporated in the models. The adjusted *R*^2^ of the linear tree model was 0.679.

As was the case with the linear models, it is evident, that content preference (CP) had the most significant effect on OLE prediction in almost all of the models described by the Eqs (19)–(22), while Envelopment ($\hat{\text{Env}}$) affects OLE to a lesser degree. As *A* was concerned, OLE for the samples that belong to Node 5 was inversely related to *A*, indicating that the more calm a music sample was (negative values of *A* ratings) and when its content was liked, then the higher its OLE rating was. OLE for the samples, that belong to Node 6 was positively related to *A*, indicating that, when the content of a sample was liked and the more aroused a subjects felt, then the higher his/hers OLE was.

## Discussion

In subsection OLE Assessment, it was shown that OLE does not further increase when the music samples were reproduced via a surround format. However, a more detailed analysis for each music sample, e.g. employing a two-way ANOVA with two independent factors (system, music sample), could perhaps reveal a significant impact of the system factor on OLE for some of the music samples. Zielinski et al. [63] showed that the perceived quality of down-mix algorithms is affected by the spatial characteristics of the stimuli. Specifically, the quality of the stimuli that had foreground spatial content both in the front and rear channels (F-F) was greatly degraded after the downmix, while for stimuli with foreground spatial content in the front channels and background spatial content in the rear channels (F-B) the differences in perceived quality were minor. This may be also the case in the context of OLE evaluation and a future research will address this issue in detail, as the amount of data obtained in this work are not sufficient for such a detailed analysis.

The adjusted *R*^2^, *RMSE* and *MAE* (see Eqs (3)–(6) were used to evaluate the overall performance of the models described in the previous section (see Table 6). It can be observed that the linear models (Model 1, 2 and 3) behaved in a very similar way and their corresponding performance metrics do not differentiate significantly. However, the proposed Linear Tree Model predicted OLE more accurately than the linear models, as is indicated by all these three metrics. To examine if there were statistically significant differences between the mean absolute errors (*MAE*) of the four implemented models, Friedman statistical test was conducted along with post hoc analysis [64, 65]. This non-parametric test was selected as the distributions of mean absolute errors strongly deviate from a normal distribution. It was found that there was a statistically significant difference in the distributions of mean absolute errors of the four proposed OLE prediction models (*χ*^2^(3, *n* = 315) = 25.29, *p* <.001). Dunn’s pairwise test were carried out using a Bonferroni correction and there were significant differences between Linear Tree Model and Model 1 (*p* <.001), Linear Tree Model and Model 2 (*p* <.001) and Linear Tree Model and Model 3 (*p* = .005). Between Model 1,2 and 3 no significant differences were found.

**Table 6:** Evaluation of the proposed OLE prediction models, reporting RMSE and MAE for the whole dataset and separately for positive and negative OLE ratings.

Models	Adjusted R^2^	RMSE	MAE	RMSE_OLE>0_	RMSE_OLE≤0_	MAE_OLE>0_	MAE_OLE≤0_
Model 1 (CP, System)	0.635	26.79	21.23 ±16.35	22.25	35.82	17.39 ±13.75	31.02 ±18.97
Model 2 ($\text{CP},\hat{\text{Env}}$)	0.619	27.41	21.69 ±16.78	22.26	36.82	17.8 ±13.84	31.47 ±19.99
Model 3 ($\text{CP},\hat{\text{Env}},\left|A\right|$)	0.626	27.11	21.33 ±16.76	22.54	36.22	17.56 ±14.02	30.91 ±19.73
Linear Tree Model	**0.679**	**25.23**	**19.31±16.27**	**20.51**	**34.43**	**15.68** ±13.17	**28.62** ±19.82

Comparative *RMSE* and *MAE* performance is also given in Table 6 for two separate user Overall Listening Experience categories: high OLE (OLE >0) and low OLE (OLE ≤0). These results indicate that low OLE ratings fail to be predicted accurately, while high OLE ratings are predicted with much higher accuracy. It is evident, that although Models 1, 2 and 3 had almost the same *RMSE* and *MAE* for both categories, the Linear Tree model was able to predict OLE ratings better for both categories and especially for the high OLE category (see *RMSE*_OLE>0_ and *MAE*_OLE>0_).

Furthermore, the prediction accuracy of the proposed model seems to outperform previously derived models predicting the Overall Listening Experience of these three spatial audio reproduction formats. Specifically, the Cragg and Uhler’s pseudo *R*^2^ of the cumulative link model proposed in [20] has a value of 0.538, considerably lower than the accuracy of all the models investigated in this research. Thus, it is evident that not only the incorporation of affective attributes enhance the prediction accuracy of the model but also the proposed methodology may be more suitable for approaching the complex problem of OLE formation in spatial audio systems.

The Linear Tree Model revealed some underlying mechanisms that contribute to OLE, based on combinations of user content preference, audio system generated envelopment and resulting arousal from the stimuli. Especially, Arousal seems to have a significant role on OLE formation, only when the content of a music sample was positively assessed. In this respect, the current work may be considered as exploratory, since arousal describes a broad variety of discrete emotions, which cannot be easily discriminated by the current procedure. Future work should investigate such limitation of the proposed models.

Although the above models cannot be generalized at this stage, the proposed methodology and analysis provides useful intuition for the way OLE is formed. Creating a larger dataset is crucial to train more accurate and higher complexity model, which could be then utilized in several applications e.g. music recommendation systems, adaptation of multichannel audio to other reproduction formats, etc, and improve audio experience by the users. From an engineering perspective, the decoding of the mechanisms that affect OLE is of high interest, as it will allow to process signal related features form audio files in order to create content and audio systems of higher preference either on a personalized level or for specific listener groups.

## Conclusion

This work has addressed the problem of individualized user Overall Listening Experience for different music reproduction formats via a 3-stage experimental procedure which provided ratings assessing separate domains of the subject’s experience. Thus, the subject’s cognitive state with respect to content was assessed via a preference rating, providing appropriate sample selection for the other test stages. The dominant perceptual effect generated by the different audio presentation was monitored via sound Envelopment and Clarity metrics and finally, the induced emotional response was rated via the Valence-Arousal (circumplex) model. Analysis of the test data, as well as the formulation of novel modeling approaches have revealed useful relationships between these assessed attributes mechanisms and OLE.

Here, the dominant attribute was found to be the prior listener familiarity F and content preference (CP), attributes which were found to be interrelated, i.e. low familiarization resulted in low CP ratings and high familiarization resulted in high CP ratings. With respect to system dependent attributes, the generated spatial Envelopment (Env) level was found to describe sufficiently the three different spatial audio reproduction formats, while a second assessed attribute, this of Clarity (Cl) was not found to differentiate strongly between stereo and surround formats. As for the emotional assessments, it was found that OLE and induced Valence (*V*) were correlated, and thus can be interpreted as describing the same attribute. For the Arousal (*A*), it was found that surround and stereo format presentations lead to more intense emotions compared to mono, as the absolute values of *A* ratings were statistically significant lower when the music samples were reproduced by only a single channel/loudspeaker.

The work examined various linear models combining ratings of these attributes in predicting OLE. Content Preference (CP) was found to be the main predictor for OLE, Envelopment (Env) had a smaller impact on predicting OLE, while absolute ratings of Arousal (|*A*|) had a minor linear effect on OLE prediction. Furthermore, a linear model was implemented, which provided good fitting between signal derived features to the envelopment listener mean ratings for each sample and its output ($\hat{\text{Env}}$) was incorporated in the proposed OLE prediction models, replacing efficiently listener’s ratings (Env).

A linear tree algorithm for OLE prediction was also proposed to obtain a deeper understanding of the way the Overall Preference is formed by groups of subjects of similar CP. Such approach splits the feature space of OLE into subspaces based on attributes values and in these subspaces, linear relationships between cognition, perception and affective states are utilized to predict OLE. This novel analysis has indicated that when the content of a music sample was greatly disliked by the subject, then CP dominated OLE, while for the other cases, both CP and Env affected significantly OLE. OLE for samples that had positive CP values and negative *A* values, were inversely related to *A*, indicating that a more calm, serene affective state resulted to higher OLE rating. Arousal was found to be proportionally related to OLE for samples with positive CP and *A* values, indicating that the more intense the excitement was, then the higher was the OLE rating, if the content of the sample was liked. Apart from such multiple level associations, the proposed Linear Model Tree resulted in more accurate prediction for the overall listener experience than simple linear modeling and thus introduces a promising framework for analysis of engineering problems addressing user’s prior preferences, system performance factors leading to perceived qualities and generated emotional responses.

## Supporting information

S1 File Music samples selection. (PDF)

S2 File Post-screening of the assessors. (PDF)

S3 File Statisical analysis. (PDF)
