# Supplementary material for: A listener preference model for spatial sound reproduction, incorporating affective response
Source: PLoS One. 2023 Jun 14;18(6):e0285135. doi: 10.1371/journal.pone.0285135 (PMC10266670; doi:10.1371/journal.pone.0285135)
Supplement: S1 File — (PDF) [file pone.0285135.s001.pdf]

S1 File

Music samples selection The music samples selected for the experimental procedure are shown in Table S1. The tracks were chosen in such a way as to represent as many different genres of music as possible, given the relative small amount of available multichannel recordings, so as our proposed methodology could account for a wide range of types of music. Special care was also given to the spatial content of the music samples. Based on the methodology introduced in [1–3], the music samples were divided in two categories: foreground-foreground (F-F) and foreground-background (F-B), based on the spatial content of the front and rear channels respectively.

Table S1. Music Tracks Employed in the Experimental Procedure

Music Tracks	Composers	Genres	Spatial Characteristic
Alexander's Entry into Pskov	Sergei Prokofiev	Classical (Choir)	F-B
All Blues	Miles Davis	Jazz	F-B
Brasileirinho	Waldir Azevedo	Ethnic	F-F
Concerto for Oboe, Violin-Adagio	J.S. Bach	Classical (orchestral)	F-B
Grave in D minor	Giuseppe Tartini	Classical (violin)	F-B
In my Life	Beatles	Pop	F-B
Money	Pink Floyd	Rock	F-F
New York Doll	Michael Hill	Blues	F-F
Pengalang	Tabuh Batel	Ethnic	F-F
Peter Gunn	Henry Mancini	Soundtrack	F-F
Piano Sonata No.4 in E flat	W.A. Mozart	Classical (piano)	F-B
Rauk, part 2	Anders Åstrand	Ethnic	F-F
Riders on the Storm	The Doors	Rock	F-F
Something She Has to Do	Philip Glass	Soundtrack	F-B
Tres movimientos tanguísticos portenos	Astor Piazzolla	Ethnic	F-F

References

1. Zielinski SK, Rumsey F, Bech S. Effects of down-mix algorithms on quality of surround sound. Journal of the Audio Engineering Society. 2003;51(9):780–798.

2. Zielinski SK, Rumsey F, Bech S. Comparison of quality degradation effects caused by limitation of bandwidth and by down-mix algorithms in consumer multichannel audio delivery systems. In: 114th AES Convention; 2003.

3. Zielinski SK, Rumsey F, Kassier R, Bech S. Comparison of basic audio quality and timbral and spatial fidelity changes caused by limitation of bandwidth and by down-mix algorithms in 5.1 surround audio systems. Journal of the Audio Engineering Society. 2005;53(3):174–192.
