# Supplementary material for: A listener preference model for spatial sound reproduction, incorporating affective response
Source: PLoS One. 2023 Jun 14;18(6):e0285135. doi: 10.1371/journal.pone.0285135 (PMC10266670; doi:10.1371/journal.pone.0285135)
Supplement: S2 File — (PDF) [file pone.0285135.s002.pdf]

S2 File

Post-screening of the assessors. During Listening Session S2, one randomly selected spatial format was presented twice, so that each subject had evaluated 10 conditions (5 music excerpts * 2 spatial quality attributes) twice. For each subject, 4 duplicate conditions were presented in surround format, 4 in stereo format and 2 in mono format. The choice for this format representation was based on an informal test, which indicated that mono format was easily detected by the subjects. Thus, the mono format should not be equally represented as stereo or surround, in order to provide a more robust and reliable result for the subject's attention and subsequent ratings. Special care was taken so that each music sample was presented for the same number of times throughout the whole listening procedure.

A measure of the reliability of the subjects is how close these evaluations are. For this purpose, a statistical tool for measuring the performance of assessors, named expertise gauge (eGauge) [1], was used. This model implements an ANOVA-based approach to quantify the reliability, discrimination and agreement of a listening panel. In this case, the metric that is mainly important is reliability. If one subject had a score lower than the rejection threshold, he/she was rejected. A total of 4 subjects were rejected and their assessments were excluded from all the three sessions of the experiment, resulting in 21 valid subjects.

References

1. Lorho G, Le Ray G, Zacharov N. eGauge—a measure of assessor expertise in audio quality evaluations. In: Audio Engineering Society Conference: 38th International Conference: Sound Quality Evaluation; 2010.
