# Supplementary material for: A listener preference model for spatial sound reproduction, incorporating affective response
Source: PLoS One. 2023 Jun 14;18(6):e0285135. doi: 10.1371/journal.pone.0285135 (PMC10266670; doi:10.1371/journal.pone.0285135)
Supplement: S3 File — (PDF) [file pone.0285135.s003.pdf]

S3 File

Statisical Analysis. In context of result analysis, the attributes in Table 1, which correspond to the responses of the participants, can be grouped into different types: categorical, ordinal, interval and ratio variables. The experiments employ mostly interval variable. Thus, parametric statistics can be applied to analyze the ratings of the participants. When measuring the linear correlation of two interval variables, Pearsons' r was used. Spearmans' ρ can be also used, if the relationship of two variables is nonlinear but monotonic, while if variables are ordinal-scaled, Kendall's τ was used. The correlation metrics described above result to values ranging from -1 to 1. A value of -1 would indicate that the two variables perfectly disagree; a value of 0 would indicate that the two variables are independent and a value of 1 would indicate that the two variables perfectly agree.

Analysis of variance (ANOVA) was used to determine if there were any statistically significant differences between the ratings' means of unrelated groups e.g. M, S and Sr loudspeaker set-up, followed by post hoc tests to capture the means difference between each possible pair of groups. If the assumption of homogeneity of variance is not violated (p-value of Levene's test is greater than .05), Tukey post hoc test is used, while if homogeneity of variance is violated, a Welch ANOVA analysis is performed along with Games-Howell post hoc test. Throughout the analysis the following effect sizes are reported: eta-squared (η^2), which measures the proportion of the variance in the dependent variable that can be explained by the variance in the groups of the independent variable, is used in the one-way ANOVA analysis. It describes only the strength of association between dependent and independent variables and not the direction, so its values ranges from 0 to 1. For the two-way ANOVA analyses partial eta-squared (${\eta_G}^2$) is reported. According to [1], an η^2 of .01 is considered as small, one of .06 as medium and one of 0.13 as large. However, η^2 and ${\eta_G}^2$ cannot explain the size of means difference between each pair of groups. To this purpose, Cohen's d is employed, which is an effect size used to describe the standardized difference between two groups. A d of .2 is considered as small, one of .5 as medium and one of .8 as large [1].

References

1. Cohen J. Statistical power analysis for the behavioral sciences. New York, USA: Psychology Press; 2009.
